# Latin American Symposium on Oncological Gastroenterology

**DOI:** 10.3332/ecancer.2013.ed23

**Published:** 2013-07-04

**Authors:** Christian Caglevic, Jorge Gallardo, María Eliana San Martín

**Affiliations:** 1 Arturo López Pérez Foundation, Santiago, Chile; 2 Chairman of the Organizing Committe (SLAGO), Alemana Clinic, Santiago, Chile; 3 Executive Director, Oncology Foundation, Santiago, Chile

The Latin American Symposium on Oncological Gastroenterology (SLAGO) took place between the the 16th and 20th of April, 2013. This important conference has been organized by the Chile Cancer Foundation every two years since 2007.

This event brings together specialists from the majority of the Latin American countries, as well as recognised professionals from the United States, Canada and Europe. The growth of this event in its short history has been enormous. When the first convention took place in 2007, there were a total of 230 participants; in this fourth edition in 2013, the number of participants surpassed 1,100. These figures and the quality of the event have made the SLAGO convention the third most important oncological gastroenterology event in the western world, after ASCO GI and the ESMO congress. Since the second version of this conference, it has been held in Viña del Mar, Chile, a beautiful coastal city on the pacific, where the event will continue to be held.

This conference brings together all the different fields that are part of oncological gastroenterology, such as medical oncologists, radiotherapists, oncological digestive surgeons, endoscopic digestive gastroenterologists, radiologists, pathologists and a panel of experts in nutrition and palliative medicine.

SLAGO, in its essence, encourages the participation and development of gastroenterology and oncology in Latin America and aims to be an instance of best practice and on-going training for the various professionals involved in this area of oncology. For this reason, as well as looking for transversality amongst the various professions in parallel, the following activities have been developed:

**Course on Pre ONS Oncology Nursing Congress**

**First Meeting of Oncology Nursing**

**First Latin American Symposium on pharmaceutical chemicals**

**Oncological Kinesiology workshops**

**Course on Nutrition and Digestive Cancer**

**Palliative medicine workshop**

**Psycho-oncology workshop**

**Colorectal Cancer Consensus**

**Oncological patient reunion**

Among the participants and attendees at this important event, the following countries can be highlighted: Germany, Argentina, Canada, Belgium, Bolivia, Brazil, USA, Spain, France, New Zealand, Peru, Mexico, Uruguay, Venezuela and Chile.

The main purpose of this convention was to discuss current therapies and diagnostics and bring together health professionals who research new treatments on a daily basis, encouraging them to share experiences and the latest treatment options for neoplasia which compromise the entire digestive system.

This Congress was chaired by Dr Jorge Gallardo of Chile, and various topics of interest were developed. The curriculum and slideshows can be downloaded from www.slago.com.

After these five days of activity, it can be concluded that both the Latin American and Caribbean participants agreed that this conference is the largest gathering of the highest impact in the region, which ranks as one of the most important and busiest in this field, and they have therefore committed themselves to spread the word and attend the next SLAGO conference in 2015.

Watch videos from the conference here

